# Risk Factors and Management of Takotsubo Cardiomyopathy

**DOI:** 10.7759/cureus.2626

**Published:** 2018-05-14

**Authors:** Sidra Khalid, Aariez Khalid, Praful Maroo

**Affiliations:** 1 Internal Medicine Residency, Fairview Hospital, Cleveland Clinic, USA; 2 Biomedical Science, University of Guelph, Guelph, CAN; 3 Cardiology, Fairview Hospital, Cleveland Clinic, USA

**Keywords:** cannabis, cardiomyopathy, takotsubo

## Abstract

Takotsubo cardiomyopathy is characterized by transient left ventricular apical ballooning, which results in temporary left ventricular dysfunction. We present a case of a 62-year-old female who presented with chest pain and shortness of breath. Her electrocardiogram was suggestive of myocardial ischemia and her troponin levels were elevated. Cardiac catheterization showed mild coronary artery disease and left ventriculography revealed severe apical hypokinesia. A diagnosis of Takotsubo cardiomyopathy was made. Her hospital stay was complicated by cardiogenic shock. One of the risk factors was cannabis use. Hence, our case highlights the management of Takotsubo cardiomyopathy and its complications, along with focus on cannabis use and its association with Takotsubo cardiomyopathy.

## Introduction

Takotsubo cardiomyopathy is prevalent in 0.7-2.5% of patients who present with symptoms suggestive of myocardial infarction. It has an in-hospital mortality of 2% [1]. The commonly associated risk factors are emotional and physical stressors. Recently, cannabis use has been associated with Takotsubo cardiomyopathy [Alliu et al., Association Between Cannabis Use and Takotsubo Cardiomyopathy, Circulation Research. 2017;121:A209]. Patients commonly present with chest pain and shortness of breath. The electrocardiogram is suggestive of myocardial ischemia and elevated troponin levels. Cardiac catheterization is important to exclude coronary artery disease, in order to diagnose Takotsubo cardiomyopathy. Treatment is based on symptoms and complications due to the cardiomyopathy. Our case will delineate the pathogenesis, risk factors, and management of Takotsubo cardiomyopathy and its complications.

## Case presentation

**Figure 1:**
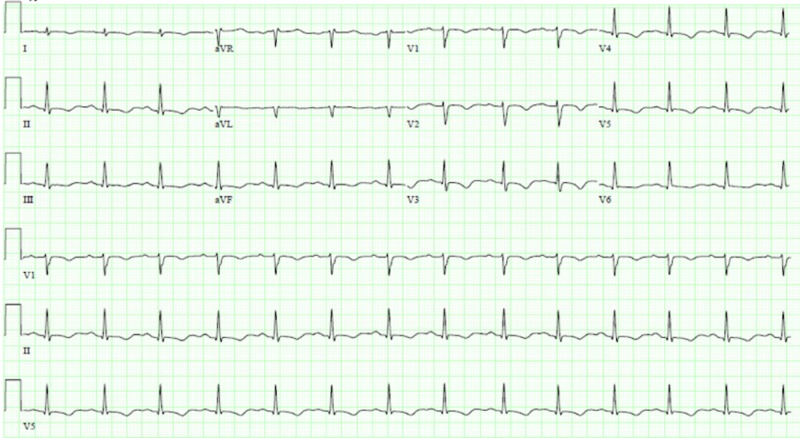
Electrocardiogram – T wave inversions in leads V2-V5, less prominent in II, III, aVF.

**Figure 2:**
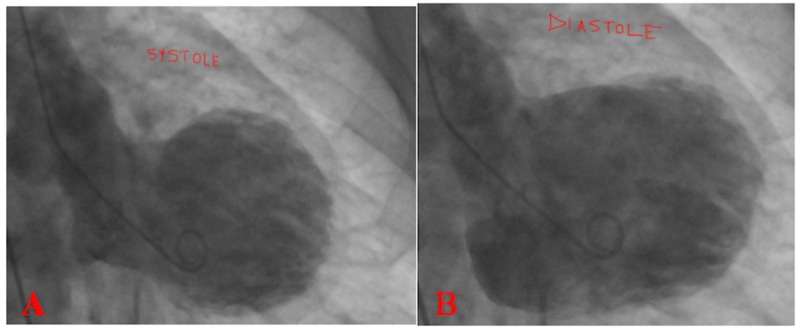
Cardiac catheterization – left ventriculography showing severe hypokinesia of the middle and apical regions of the left ventricle when comparing systole (A) and diastole (B).

A 62-year-old female presented to the emergency department with chest pain and shortness of breath. Her past medical history was significant for hyperlipidemia, hypertension, and type 2 diabetes mellitus. A family history of coronary artery disease was present. She had recent stressors at home. Her vitals were as follows: temperature 97.6 °F, blood pressure 122/95 mmHg, heart rate 76 beats/min, respiratory rate 18/min, and SpO2 93% on room air. Her physical examination was unremarkable. Her urine drug screen was positive for opiates, benzodiazepines, and tetrahydrocannabinol. Her troponin level was 0.655 ng/mL. An initial electrocardiogram showed sinus tachycardia. She was started on intravenous nitroglycerin and beta blocker. However, she became hypotensive, 84/42 mmHg, and was given intravenous fluids and started on a norepinephrine infusion. Her repeat electrocardiogram showed T wave inversions in leads V2-V5, less prominent in II, III, aVF, suggestive of myocardial ischemia (Figure 1). She was taken for cardiac catheterization immediately. Cardiac catheterization revealed mild coronary artery disease and severe apical hypokinesia with a left ventricle ejection fraction of 25-30% (Figures 2A-2B). These findings were suggestive of Takotsubo cardiomyopathy.

For cardiogenic shock, an intra-aortic balloon pump was placed. She was started on carvedilol, captopril, and heparin infusion. She became hypotensive again and was started on dopamine infusion, and carvedilol and captopril were stopped. She developed pulmonary edema, with small right-sided pleural effusion and bilateral interstitial opacities on the chest X-ray, and she was started on furosemide. Echocardiogram showed a left ventricular ejection fraction of 30% with akinesia of the mid and distal anterior wall and septum, entire apex, mid and distal inferior wall, and mid anterolateral and mid inferoseptal segments. She continued to improve and the intra-aortic balloon pump was removed and dopamine was stopped. Her heparin infusion was transitioned to warfarin to prevent a left ventricular thrombus from forming. Statin was not started due to previous intolerance. She was gradually started on low dose carvedilol and lisinopril. She continued to improve and was discharged home. On a follow-up visit after two weeks, her symptoms had improved. Repeat echocardiogram showed left ventricular ejection fraction of 45% and hypokinesia of mid and distal anterior septum, apical lateral and anterior segments, and apex.

## Discussion

Takotsubo cardiomyopathy occurs in about 0.2% of all hospitalizations in the United States. It is characterized by a transient systolic and diastolic left ventricular dysfunction [2]. There is apical ballooning of the left ventricle [2]. It is thought that the underlying pathogenesis is excess catecholamines, coronary spasm, coronary microvascular dysfunction, or myocarditis [3]. The catecholamine-mediated effects on the heart result in structural changes such as increased extracellular matrix, contraction band necrosis, and mild neutrophilic infiltration. Catecholamines also increase the levels of reactive oxygen species and profibrotic angiotensin II. Catecholamines also activate transforming growth factor B that stimulates extracellular matrix proteins, resulting in myocardial disarray. Elevated levels of catecholamines stimulate B-adrenoceptors and alter the calcium regulatory system, leading to slowed cardiomyocyte relaxation and impaired cardiac function. When the B2-adrenergic receptors are activated by epinephrine at higher concentrations, there is a negative inotropic effect on myocyte contraction; however, there is simultaneous activation of the phosphatidylinositol 3-kinase and protein kinase B (PI3K-AKT) pathway. The PI3K-AKT pathway provides a favorable outcome as it decreases the myocardial disarray associated with excess catecholamines [1].

The associated risk factors in a study by Deshmukh et al. were age > 55 years, smoking, alcohol abuse, anxiety states, and hyperlipidemia [4]. It is a condition which commonly affects elderly females and is associated with an emotional or a physical trigger [2]. The diagnostic criteria by the Mayo clinic states suspicion of acute myocardial infarction based on precordial pain and ST elevation on the acute electrocardiogram; transient hypokinesia or akinesia of the middle and apical regions of the left ventricle on ventriculography or echocardiography; normal coronary arteries on cardiac catheterization within 24 hrs of symptom onset; absence of recent significant head injury, intracranial hemorrhage, suspicion of pheochromocytoma, myocarditis, or hypertrophic cardiomyopathy [3].

The initial clinical features are similar to patients presenting with an acute coronary syndrome with chest pain and dyspnea. Initial investigations, such as elevated troponin levels and an electrocardiogram suggestive of myocardial ischemia are present. Therefore, cardiac catheterization is important to exclude underlying coronary artery disease. Takotsubo cardiomyopathy also results in acute heart failure, which is seen with elevated proBNP levels and increased left ventricular end diastolic pressure. It can also lead to complications, such as stroke and death. In a study by Templin et al. rate of death was 5.6% and rate of stroke or transient ischemic attack was 1.7% [2]. Other complications include hypotension, ventricular rupture, thrombosis of the left ventricular apex and torsade de pointes. Management initially depends on the patient’s clinical condition. If there is hemodynamic instability then placement of an intra-aortic balloon pump is beneficial, along with vasopressors. Hemodynamic patients are treated with beta blockers, angiotension converting enzyme inhibitors, and anticoagulation [3].

Additionally, cannabinoid use is another proposed risk factor for Takotsubo cardiomyopathy [5,6]. It is suggested that endocannabinoids act directly and on cannabinoid-1 (CB-1) receptors leading to cardiovascular effects, such as reduced contractility [4]. Likewise, exogenous cannabis causes tachycardia acutely, bradycardia and hypotension chronically, and with further use it could impair myocardial function [6]. A study by Alliu et al. concluded that nondependent cannabis use was associated with significantly increased odds of Takotsubo cardiomyopathy [Alliu, 2017]. Another study by Singh et al. concluded marijuana use is linked to transient left ventricular regional ballooning in younger individuals and is associated with significant morbidity [Singh et al., Marijuana Use is an Independent Predictor of Stress Cardiomyopathy in Younger Men, Circulation. 2016;134:A14100].

## Conclusions

Our case highlights the management of Takotsubo cardiomyopathy and its complications. It also states the proposed pathogenesis and risk factors, especially cannabis use, which has been implicated as a risk factor in recent studies as well.
